# 2-Amino-6-(dimethyl­amino)pyridine-3,5-dicarbonitrile

**DOI:** 10.1107/S1600536812017278

**Published:** 2012-04-25

**Authors:** Shaaban K. Mohamed, Ahmed M. Soliman, Eman M. M. Abdel-Raheem, Sohail Saeed, Wing-Tak Wong

**Affiliations:** a Chemistry and Environmental Division, Manchester Metropolitan University, Manchester, M1 5GD, England; b Department of Chemistry, Faculty of Science, Sohag University, Egypt; c Department of Chemistry, Research Complex, Allama Iqbal Open University, Islamabad 44000, Pakistan; d Department of Chemistry, The University of Hong Kong, Pokfulam Road, Pokfulam, Hong Kong SAR, People’s Republic of China

## Abstract

The title compound, C_9_H_9_N_5_, is slightly twisted from planarity, with a maximum deviation of 0.0285 (13) Å from the pyridine plane for the C atom bearing the amino group. The cyano groups are on different sides of the pyridine plane, with C- and N-atom deviations of 0.072 (3)/0.124 (4) and −0.228 (4)/−0.409 (5) Å from the pyridine plane. In the crystal, N—H⋯N and C—H⋯N hydrogen bonds connect the mol­ecules into zigzag chains running along the *c* axis.

## Related literature

For the synthesis of similar structures, see: Horton *et al.* (2012*a* ▶,*b* ▶); Soliman *et al.* (2012 ▶). For the biological significance of cyano­amino pyridines, see: Al-Haiza *et al.* (2003 ▶); Bhalerao & Krishnaiah (1995 ▶); Deo *et al.* (1990 ▶); Murata *et al.* (2003 ▶); Konda *et al.* (2010 ▶); Altomare *et al.* (2000 ▶); Hosni & Abdulla (2008 ▶); Shishoo *et al.* (1983 ▶).
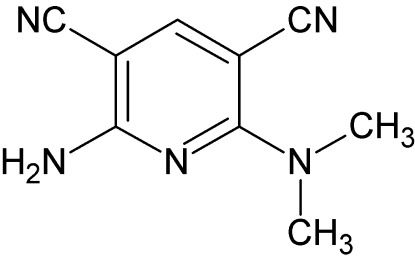


## Experimental

### 

#### Crystal data

C_9_H_9_N_5_
*M* *_r_* = 187.21
Monoclinic, 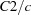

*a* = 28.667 (7) Å
*b* = 3.9702 (10) Å
*c* = 17.950 (4) Å
β = 112.920 (3)°
*V* = 1881.7 (8) Å^3^
*Z* = 8
Mo *K*α radiation
μ = 0.09 mm^−1^
*T* = 296 K
0.32 × 0.21 × 0.03 mm

#### Data collection

Bruker SMART 1000 CCD diffractometer
Absorption correction: multi-scan (*SADABS*; Sheldrick, 2004 ▶) *T*_min_ = 0.972, *T*_max_ = 0.997
4846 measured reflections
1658 independent reflections
1173 reflections with *I* > 2σ(*I*)
*R*_int_ = 0.025

#### Refinement

*R*[*F* ^2^ > 2σ(*F* ^2^)] = 0.048
*wR*(*F* ^2^) = 0.147
*S* = 1.03
1658 reflections
138 parameters
H atoms treated by a mixture of independent and constrained refinement
Δρ_max_ = 0.16 e Å^−3^
Δρ_min_ = −0.19 e Å^−3^

### 

Data collection: *SMART* (Bruker, 1998 ▶); cell refinement: *SAINT* (Bruker, 2006 ▶); data reduction: *SAINT*; program(s) used to solve structure: *SHELXS97* (Sheldrick, 2008 ▶); program(s) used to refine structure: *SHELXL97* (Sheldrick, 2008 ▶); molecular graphics: *Mercury* (Macrae *et al.*, 2008 ▶); software used to prepare material for publication: *SHELXL97*.

## Supplementary Material

Crystal structure: contains datablock(s) global, I. DOI: 10.1107/S1600536812017278/im2369sup1.cif

Structure factors: contains datablock(s) I. DOI: 10.1107/S1600536812017278/im2369Isup2.hkl

Supplementary material file. DOI: 10.1107/S1600536812017278/im2369Isup3.cml

Additional supplementary materials:  crystallographic information; 3D view; checkCIF report

## Figures and Tables

**Table 1:** Hydrogen-bond geometry (Å, °)

*D*—H⋯*A*	*D*—H	H⋯*A*	*D*⋯*A*	*D*—H⋯*A*
N2—H1⋯N3^i^	0.88 (3)	2.25 (3)	3.119 (3)	167 (2)
N2—H2⋯N1^ii^	0.88 (3)	2.43 (3)	3.260 (3)	158 (3)
C3—H3⋯N4^iii^	0.93	2.55	3.471 (4)	170

Symmetry codes: (i) 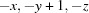; (ii) 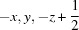; (iii) 
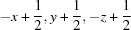
.

**Fig. 1.**
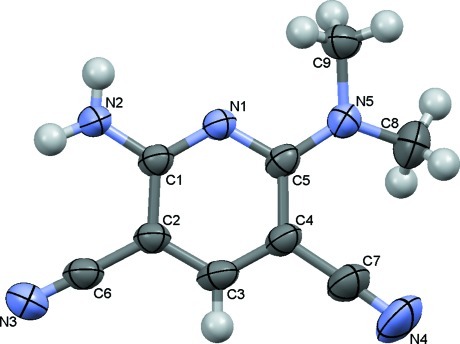
Molecular structure of the title compound showing thermal ellipsoids on the 50% probability level.

## supplementary crystallographic information

### Comment

In continuation of our research interest in the synthesis of potential biologically active molecules (Soliman *et al.*, 2012; Horton *et al.*, 2012*a*,*b*), we got prompted to study the chemical and pharmacological characterization of new cyano-amino pyridine derivatives due to their vibrant chemical activities. Hence cyano-amino pyridines have been considered as convenient synthons due to their diverse applications particularly in organic synthesis (Shishoo *et al.*, 1983; Deo *et al.*, 1990; Bhalerao & Krishnaiah, 1995; Al-Haiza *et al.*, 2003) and medicinal chemistry (Altomare *et al.*, 2000; Hosni & Abdulla, 2008; Murata *et al.*, 2003; Konda *et al.*, 2010).

The title compound, 2-amino-6-(dimethylamino)-pyridine-3,5-dicarbonitrile, is slightly twisted. The maximum deviation from the mean plane of the pyridyl ring, N1/C1—C5 (marked with asterisk) is 0.0285 (13) Angstrom. The cyano groups are flipped to different sides of the pyridine plane with atoms C6 & N3 showing deviations of +0.072 (3) Å and +0.124 (4) Å, while atoms C7 & N4 are bent out of the pyridine plane by -0.228 (4) Å and -0.409 (5) Å, respectively.

Hydrogen bonding interactions are observed in the crystal lattice connecting the molecules into zigzag chains running along the *c*-axis. As it is expected, N—H···N interactions are shorter as the observed N4···H3(–C3) distance.

### Experimental

The title compound (1) was obtained as a by-product from the reaction of 2-amino-6-chloropyridine-3,5-dicarbonitrile (1 mmol; 179 mg) with amino guanidine (1 mmol; 74 mg) in dimethylformamide. The reaction mixture was refluxed for 4 h at 426 K and then poured on cold water. A solid product was filtered off, dried and recrystallized from ethanol to afford cupric needles which were suitable for X-Ray diffraction without further recrystallization. Yield 45% and m.p. 453 K.

### Refinement

The structure was solved by direct methods (*SHELXS97*, Sheldrick, 2008) and expanded using Fourier techniques. All non-H atoms were refined anisotropically.

C-bound H atoms are all placed at geometrical positions with C—H = 0.93 and 0.96 Å for phenyl and methyl H-atoms, respectively. C-bound phenyl hydrogen atoms are refined using a riding model with *U*_iso_(H) = 1.2*U*_eq_(C), methyl H-atoms are refined using a riding model with *U*_iso_(H) = 1.5*U*_eq_(C). N-bound H atoms were located from the difference Fourier map and were refined isotropically.

Highest peak is 0.16 at (0.1967, 0.2214, 0.0041) [1.04 Å from H8C] Deepest hole is -0.19 at (0.1226, 0.1471, 0.2613) [1.01 Å from C5]

### Crystal data

**Table:**

C_9_H_9_N_5_	*F*(000) = 784
*M**_r_* = 187.21	*D*_x_ = 1.322 Mg m^−^^3^
Monoclinic, *C*2/*c*	Mo *K*α radiation, λ = 0.71073 Å
Hall symbol: -C 2yc	Cell parameters from 4846 reflections
*a* = 28.667 (7) Å	θ = 3.1–25.0°
*b* = 3.9702 (10) Å	µ = 0.09 mm^−^^1^
*c* = 17.950 (4) Å	*T* = 296 K
β = 112.920 (3)°	Plate, yellow
*V* = 1881.7 (8) Å^3^	0.32 × 0.21 × 0.03 mm
*Z* = 8	

### Data collection

**Table:**

Bruker SMART 1000 CCD diffractometer	1658 independent reflections
Radiation source: fine-focus sealed tube	1173 reflections with *I* > 2σ(*I*)
Graphite monochromator	*R*_int_ = 0.025
ω and φ scans	θ_max_ = 25.0°, θ_min_ = 3.1°
Absorption correction: multi-scan (*SADABS*; Sheldrick, 2004)	*h* = −33→34
*T*_min_ = 0.972, *T*_max_ = 0.997	*k* = −4→4
4846 measured reflections	*l* = −21→18

### Refinement

**Table:**

Refinement on *F*^2^	Primary atom site location: structure-invariant direct methods
Least-squares matrix: full	Secondary atom site location: difference Fourier map
*R*[*F*^2^ > 2σ(*F*^2^)] = 0.048	Hydrogen site location: inferred from neighbouring sites
*wR*(*F*^2^) = 0.147	H atoms treated by a mixture of independent and constrained refinement
*S* = 1.03	*w* = 1/[σ^2^(*F*_o_^2^) + (0.0915*P*)^2^ + 0.1839*P*] where *P* = (*F*_o_^2^ + 2*F*_c_^2^)/3
1658 reflections	(Δ/σ)_max_ < 0.001
138 parameters	Δρ_max_ = 0.16 e Å^−^^3^
0 restraints	Δρ_min_ = −0.19 e Å^−^^3^

### Special details

**Table:**

Geometry. All e.s.d.'s (except the e.s.d. in the dihedral angle between two l.s. planes) are estimated using the full covariance matrix. The cell e.s.d.'s are taken into account individually in the estimation of e.s.d.'s in distances, angles and torsion angles; correlations between e.s.d.'s in cell parameters are only used when they are defined by crystal symmetry. An approximate (isotropic) treatment of cell e.s.d.'s is used for estimating e.s.d.'s involving l.s. planes.
Refinement. Refinement of *F*^2^ against ALL reflections. The weighted *R*-factor *wR* and goodness of fit *S* are based on *F*^2^, conventional *R*-factors *R* are based on *F*, with *F* set to zero for negative *F*^2^. The threshold expression of *F*^2^ > σ(*F*^2^) is used only for calculating *R*-factors(gt) *etc*. and is not relevant to the choice of reflections for refinement. *R*-factors based on *F*^2^ are statistically about twice as large as those based on *F*, and *R*- factors based on ALL data will be even larger.

### Fractional atomic coordinates and isotropic or equivalent isotropic displacement parameters (Å^2^)

**Table:**

	*x*	*y*	*z*	*U*_iso_*/*U*_eq_	
N1	0.07712 (6)	0.0877 (4)	0.27256 (9)	0.0417 (5)	
N2	0.00997 (6)	0.2230 (6)	0.15638 (13)	0.0573 (6)	
H1	−0.0050 (9)	0.284 (6)	0.1053 (17)	0.068 (8)*	
H2	−0.0096 (12)	0.128 (8)	0.1777 (19)	0.094 (9)*	
N3	0.05670 (8)	0.6406 (6)	0.02533 (12)	0.0669 (6)	
N4	0.25896 (8)	0.0677 (9)	0.35083 (15)	0.1042 (10)	
N5	0.14100 (6)	−0.0606 (5)	0.39210 (10)	0.0488 (5)	
C1	0.06018 (7)	0.2187 (5)	0.19854 (12)	0.0415 (5)	
C2	0.09339 (7)	0.3580 (5)	0.16489 (12)	0.0431 (5)	
C3	0.14482 (7)	0.3269 (6)	0.20982 (13)	0.0499 (6)	
H3	0.1676	0.4079	0.1887	0.060*	
C4	0.16300 (7)	0.1796 (5)	0.28473 (13)	0.0463 (5)	
C5	0.12699 (7)	0.0699 (5)	0.31732 (12)	0.0413 (5)	
C6	0.07369 (8)	0.5132 (6)	0.08746 (14)	0.0498 (6)	
C7	0.21622 (9)	0.1175 (7)	0.32283 (15)	0.0673 (7)	
C8	0.18827 (9)	0.0208 (8)	0.45832 (15)	0.0782 (8)	
H8A	0.1828	0.0356	0.5077	0.117*	
H8B	0.2006	0.2328	0.4478	0.117*	
H8C	0.2127	−0.1520	0.4634	0.117*	
C9	0.10351 (8)	−0.2200 (6)	0.41700 (13)	0.0561 (6)	
H9A	0.1197	−0.3882	0.4572	0.084*	
H9B	0.0778	−0.3238	0.3710	0.084*	
H9C	0.0884	−0.0530	0.4392	0.084*	

### Atomic displacement parameters (Å^2^)

**Table:**

	*U*^11^	*U*^22^	*U*^33^	*U*^12^	*U*^13^	*U*^23^
N1	0.0341 (9)	0.0539 (10)	0.0397 (10)	−0.0009 (7)	0.0172 (7)	−0.0014 (8)
N2	0.0344 (10)	0.0948 (16)	0.0427 (12)	−0.0056 (9)	0.0150 (9)	0.0117 (11)
N3	0.0644 (13)	0.0872 (15)	0.0559 (13)	−0.0010 (11)	0.0308 (11)	0.0131 (12)
N4	0.0412 (12)	0.175 (3)	0.0971 (19)	0.0216 (15)	0.0270 (12)	−0.0016 (19)
N5	0.0376 (9)	0.0639 (11)	0.0435 (10)	0.0045 (8)	0.0142 (8)	0.0037 (9)
C1	0.0364 (10)	0.0501 (12)	0.0411 (12)	−0.0021 (8)	0.0186 (9)	−0.0048 (9)
C2	0.0404 (11)	0.0522 (12)	0.0425 (12)	−0.0026 (9)	0.0224 (9)	−0.0020 (10)
C3	0.0419 (12)	0.0610 (13)	0.0572 (14)	−0.0054 (9)	0.0307 (11)	−0.0060 (11)
C4	0.0332 (11)	0.0580 (13)	0.0515 (13)	0.0009 (9)	0.0205 (9)	−0.0041 (11)
C5	0.0348 (10)	0.0455 (11)	0.0449 (12)	0.0010 (8)	0.0169 (9)	−0.0067 (9)
C6	0.0468 (12)	0.0611 (14)	0.0509 (14)	−0.0040 (10)	0.0292 (11)	−0.0023 (12)
C7	0.0445 (14)	0.0956 (19)	0.0682 (17)	0.0067 (12)	0.0288 (12)	−0.0019 (14)
C8	0.0492 (14)	0.108 (2)	0.0642 (17)	0.0041 (14)	0.0074 (12)	0.0105 (16)
C9	0.0532 (13)	0.0662 (14)	0.0575 (14)	0.0078 (11)	0.0310 (12)	0.0106 (12)

### Geometric parameters (Å, º)

**Table:**

N1—C1	1.330 (2)	C2—C6	1.421 (3)
N1—C5	1.341 (2)	C3—C4	1.370 (3)
N2—C1	1.340 (3)	C3—H3	0.9300
N2—H1	0.88 (3)	C4—C7	1.429 (3)
N2—H2	0.88 (3)	C4—C5	1.438 (3)
N3—C6	1.146 (3)	C8—H8A	0.9600
N4—C7	1.146 (3)	C8—H8B	0.9600
N5—C5	1.346 (3)	C8—H8C	0.9600
N5—C8	1.449 (3)	C9—H9A	0.9600
N5—C9	1.459 (3)	C9—H9B	0.9600
C1—C2	1.423 (3)	C9—H9C	0.9600
C2—C3	1.383 (3)		
			
C1—N1—C5	120.56 (16)	C7—C4—C5	123.7 (2)
C1—N2—H1	124.9 (15)	N1—C5—N5	116.88 (16)
C1—N2—H2	118 (2)	N1—C5—C4	120.45 (18)
H1—N2—H2	116 (3)	N5—C5—C4	122.66 (18)
C5—N5—C8	123.56 (19)	N3—C6—C2	178.3 (2)
C5—N5—C9	120.21 (17)	N4—C7—C4	177.7 (3)
C8—N5—C9	114.24 (18)	N5—C8—H8A	109.5
N1—C1—N2	117.60 (18)	N5—C8—H8B	109.5
N1—C1—C2	122.11 (18)	H8A—C8—H8B	109.5
N2—C1—C2	120.28 (19)	N5—C8—H8C	109.5
C3—C2—C6	122.45 (17)	H8A—C8—H8C	109.5
C3—C2—C1	117.08 (19)	H8B—C8—H8C	109.5
C6—C2—C1	120.45 (17)	N5—C9—H9A	109.5
C4—C3—C2	121.48 (18)	N5—C9—H9B	109.5
C4—C3—H3	119.3	H9A—C9—H9B	109.5
C2—C3—H3	119.3	N5—C9—H9C	109.5
C3—C4—C7	118.05 (19)	H9A—C9—H9C	109.5
C3—C4—C5	118.02 (18)	H9B—C9—H9C	109.5

### Hydrogen-bond geometry (Å, º)

**Table:**

*D*—H···*A*	*D*—H	H···*A*	*D*···*A*	*D*—H···*A*
N2—H1···N3^i^	0.88 (3)	2.25 (3)	3.119 (3)	167 (2)
N2—H2···N1^ii^	0.88 (3)	2.43 (3)	3.260 (3)	158 (3)
C3—H3···N4^iii^	0.93	2.55	3.471 (4)	170

Symmetry codes: (i) −*x*, −*y*+1, −*z*; (ii) −*x*, *y*, −*z*+1/2; (iii) −*x*+1/2, *y*+1/2, −*z*+1/2.
